# Non-typhoidal *Salmonella* serovars associated with invasive and non-invasive disease in the Lao People’s Democratic Republic

**DOI:** 10.1093/trstmh/trx076

**Published:** 2018-01-12

**Authors:** Tu Le Thi Phuong, Sayaphet Rattanavong, Manivanh Vongsouvath, Viengmon Davong, Nguyen Phu Huong Lan, James I Campbell, Thomas C Darton, Guy E Thwaites, Paul N Newton, David A B Dance, Stephen Baker

**Affiliations:** 1 Hospital for Tropical Diseases, Wellcome Trust Major Overseas Programme, Oxford University Clinical Research Unit, 764 Vo Van Kiet, Quan 5, Ho Chi Minh City, Vietnam; 2 Lao-Oxford-Mahosot Hospital-Wellcome Trust Research Unit (LOMWRU), Microbiology Laboratory, Mahosot Hospital, Vientiane Capital, Lao People’s Democratic Republic; 3 Hospital for Tropical Diseases, Ho Chi Minh City, Vietnam; 4 Centre for Tropical Medicine and Global Health, Nuffield Department of Medicine, Old Road Campus, University of Oxford, Oxford, UK; 5 Department of Infection, Immunity and Cardiovascular Disease, University of Sheffield Medical School, Sheffield, UK; 6 Faculty of Infectious and Tropical Diseases, London School of Hygiene and Tropical Medicine, London, UK; 7 Department of Medicine, University of Cambridge, Cambridge, UK

**Keywords:** Antimicrobial susceptibility, Bloodstream infections, HIV, Lao, Non-typhoidal *Salmonella*, NTS, *Salmonella* Typhimurium

## Abstract

**Background:**

Invasive non-typhoidal *Salmonella* (iNTS) disease is a well-described cause of mortality in children and human immunodeficiency virus (HIV)-infected adults in sub-Saharan Africa. Additionally, there is an ill-defined burden of iNTS disease in Southeast Asia.

**Methods:**

Aiming to investigate the causative serovars of non-invasive and iNTS disease and their associated antimicrobial susceptibility profiles in the Lao People’s Democratic Republic, we performed multilocus sequence typing and antimicrobial susceptibility profiling on 168 NTS (63 blood and 105 faecal) organisms isolated in Lao between 2000 and 2012.

**Results:**

Six different serovars were isolated from blood; *Salmonella enterica* serovar Enteritidis (n=28), *S. enterica* serovar Typhimurium (n=19) and *S. enterica* serovar Choleraesuis (n=11) accounted for >90% (58/63) of the iNTS disease cases. In contrast, the isolates from diarrhoeal faeces were comprised of 18 different serovars, the mostly commonly identified being *S. enterica* Typhimurium (n=28), *S. enterica* Weltevreden (n=14) and *S. enterica* Stanley (n=15). *S. enterica* Enteritidis and *S. enterica* Choleraesuis were significantly more associated with systemic disease than diarrhoeal disease in this patient group (p<0.001).

**Conclusions:**

We find a differing distribution of *Salmonella* sequence types/serovars between those causing iNTS disease and non-invasive disease in Lao. We conclude that there is a small but not insignificant burden of iNTS disease in Lao. Further clinical and epidemiological investigations are required to assess mortality and the role of comorbidities such as HIV.

## Introduction

Gastroenteritis caused by non-typhoidal *Salmonella* (NTS) is of significant public health importance, resulting in an estimated 94 million cases and 155 000 deaths worldwide each year.^1^ While NTS disease in humans is classically associated with self-limiting diarrhoea, these organisms may also cause invasive disease (iNTS), particularly in those who are immunocompromised due to extremes of age, malnutrition, human immunodeficiency virus (HIV) infection or malaria.^2–4^ In individuals with these risk factors living in sub-Saharan Africa, iNTS disease is a significant problem, with associated mortality rates of 20–25%.^5^ Management is further complicated by the emergence of resistance to all major classes of antimicrobial agents.^3,6^ In contrast to typical diarrhoea-causing NTS, which is thought to be transmitted by ingestion of contaminated food or contact with animals, there is some evidence from sub-Saharan Africa that human-to-human transmission of iNTS disease-causing organisms may be a more important route of infection.^7^

There are >2500 different *Salmonella* serovars that have described through structural variation in the O (lipopolysaccharide) and H (flagella) antigens. The majority of serovars associated with NTS disease are classified within *Salmonella enterica* subspecies I, a broad species encompassing organisms such as *Salmonella enterica* serovar Typhimurium and *S*. *enterica* Enteritidis, which have the ability to colonize a wide range of animal hosts and cause human disease. Subspecies I also contains more host-restricted serovars, such as *S. enterica* Gallinarum (chickens) and *S*. *enterica* Choleraesuis (pigs), which are adapted to their specific host type and rarely infect humans.^8^ It has been shown that a wide range of NTS serovars (including *S. enterica* Typhimurium and *S*. *enterica* Enteritidis) circulate on farms in Southeast Asia and much attention has been focused on sampling pigs and poultry in the region.^9^ Furthermore, less common NTS serovars are also known to circulate in Southeast Asia.^10^ A high prevalence of *S*. *enterica* Weltevreden has been reported in fish farms in Thailand and the Mekong Delta region of southern Vietnam,^11,12^ and this serovar has also recently been identified as a major cause of *Salmonella*-associated diarrhoea in the Vietnamese population.^13^

While there are considerable data regarding the circulation of NTS in animals/meat in Southeast Asia,^14,15^ there are less data regarding the serovars that cause human disease, especially from the Lao Peoples Democratic Republic (Lao), where data are scarce due to the lack of diagnostic microbiology facilities.^16^ Here we aimed to investigate the serovars causing invasive and non-invasive NTS disease and their associated antimicrobial susceptibility profiles over a 12 y period in a major tertiary hospital in Vientiane, Lao. Lao is a sparsely populated, land-locked country in Southeast Asia. This cross-sectional study represents the largest comparison of *Salmonella* associated with diarrhoea and iNTS disease in this country to date.

## Materials and methods

iNTS disease-causing isolates were collected from blood cultures submitted to the Microbiology Laboratory of Mahosot Hospital, Vientiane, Lao PDR between 2006 and 2012 as part of an ongoing fever aetiology study. Patients of any age presenting with a temperature >37.5°C for <1 month were eligible for participation. Written informed consent was provided by all patients or by a parent or guardian if <15 y of age or unable to consent because of illness. As part of the diagnostic workup, blood (10 mL from adults, 4 mL from children and 2 mL from infants) was inoculated into blood culture broths (Pharmaceutical Factory Number 2, Vientiane, Lao) and processed as previously described.^16^

All 105 faecal *Salmonella* isolates were collected from patients with diarrhoeal disease at Mahosot Hospital between 2006 and 2012, the majority of whom were admitted. Faecal culture and bacterial identification were conducted as a component of routine clinical diagnostics when requested by an attending clinician as part of their clinical investigation. Faecal specimens were inoculated onto MacConkey agar (MC; Oxoid, Basingstoke, UK) and *Salmonella-Shigella* agar (SS; Oxoid) and incubated at 37°C for 18–24 h. In addition, selenite broths were inoculated and subcultured onto MC and SS agars after 24 h of incubation.

After subculture, *Salmonella* were identified by phenotypic appearance on SS and MC agars (colourless non-lactose fermenting) and confirmed serologically by agglutination with polyvalent O antiserum (Omni-O, Bio-Rad Laboratories, Hercules, CA, USA) and biochemically by API20E (BioMerieux, Marcy l’Etoile, France). Additionally, Vi, O9 and H-d antisera were also used to further identify *S. enterica* Typhi from blood samples. Over the corresponding period we isolated 287 *S*. *enterica* Typhi and 18 *S. enterica* Paratyphi A; these were excluded from the analyses. Where possible, isolates were identified to the group level using the Wellcolex Colour *Salmonella* kit (Oxoid). All isolates were stored at −80°C using the Protect Microorganism Preservation System (Technical Service Consultants, Heywood, UK) in Vientiane prior to subculture and shipment to Vietnam for further characterization.

Antimicrobial susceptibility testing (AST) was performed using a disk diffusion method on Mueller–Hinton agar at the time of isolation and confirmed subsequently in Vietnam. AST was performed against ampicillin (10 μg), amoxicillin/clavulanate (30 μg), ceftazidime (30 μg), ceftriaxone (30 μg), chloramphenicol (30 μg), ciprofloxacin (5 μg), gentamicin (10 μg), nalidixic acid (30 μg), ofloxacin (5 μg) and trimethoprim-sulfamethoxazole (1.25/23.75 μg; all disks Thermo Fisher Scientific, Waltham, MA, USA). Susceptibility to antimicrobials was interpreted using Clinical and Laboratory Standards Institute recommendations^17^; for the purposes of the analysis presented here, organisms were grouped as susceptible and non-susceptible, with intermediate resistance included as non-susceptible. Multidrug resistance (MDR) was defined as an organism testing non-susceptible to at least three different classes of antimicrobials.

To further characterize the NTS isolates, all organisms were subjected to genotyping and molecular serotyping using multilocus sequence typing (MLST) according to previously described methods.^18^ Briefly, *Salmonella* were grown overnight on nutrient agar and total genomic DNA was extracted using Wizard Genomic DNA extraction kit (Promega, Madison, WI, USA). Seven housekeeping genes (*aroC, dnaN, hemD, hisD, purE, sucA* and *thrA*, with primer sequences accessed from an MLST database [http://mlst.warwick.ac.uk/mlst/dbs/Senterica]) were polymerase chain reaction (PCR) amplified using template genomic DNA extracted from each isolate. PCR amplicons were cleaned using Agentcourt Ampure XP (Beckman Coulter, Brea, CA, USA) and sequenced in both directions using BigDye Terminator v3 (Applied Biosystems, Waltham, MA, USA) followed by capillary sequencing on a 3130XL Genetic Analyzer (Applied Biosystems). All sequences were manually trimmed, aligned to a reference sequence and submitted to the MLST database (detailed above) for allelic profiling and molecular serotyping. A minimum spanning tree (describing the variation in the number of alleles between isolates of the seven housekeeping genes) was created with the allelic profiles using Bionumerics software (Applied Maths, Sint-Martens-Latem, Belgium).

Clinical and laboratory data were collated and analysed, with between-groups comparisons performed using Fisher’s exact test. All statistical analyses were performed using Stata version 11 (StataCorp, College Station, TX, USA). A p*-*value <0.05 was considered to be significant.

## Results

**Figure 1.**
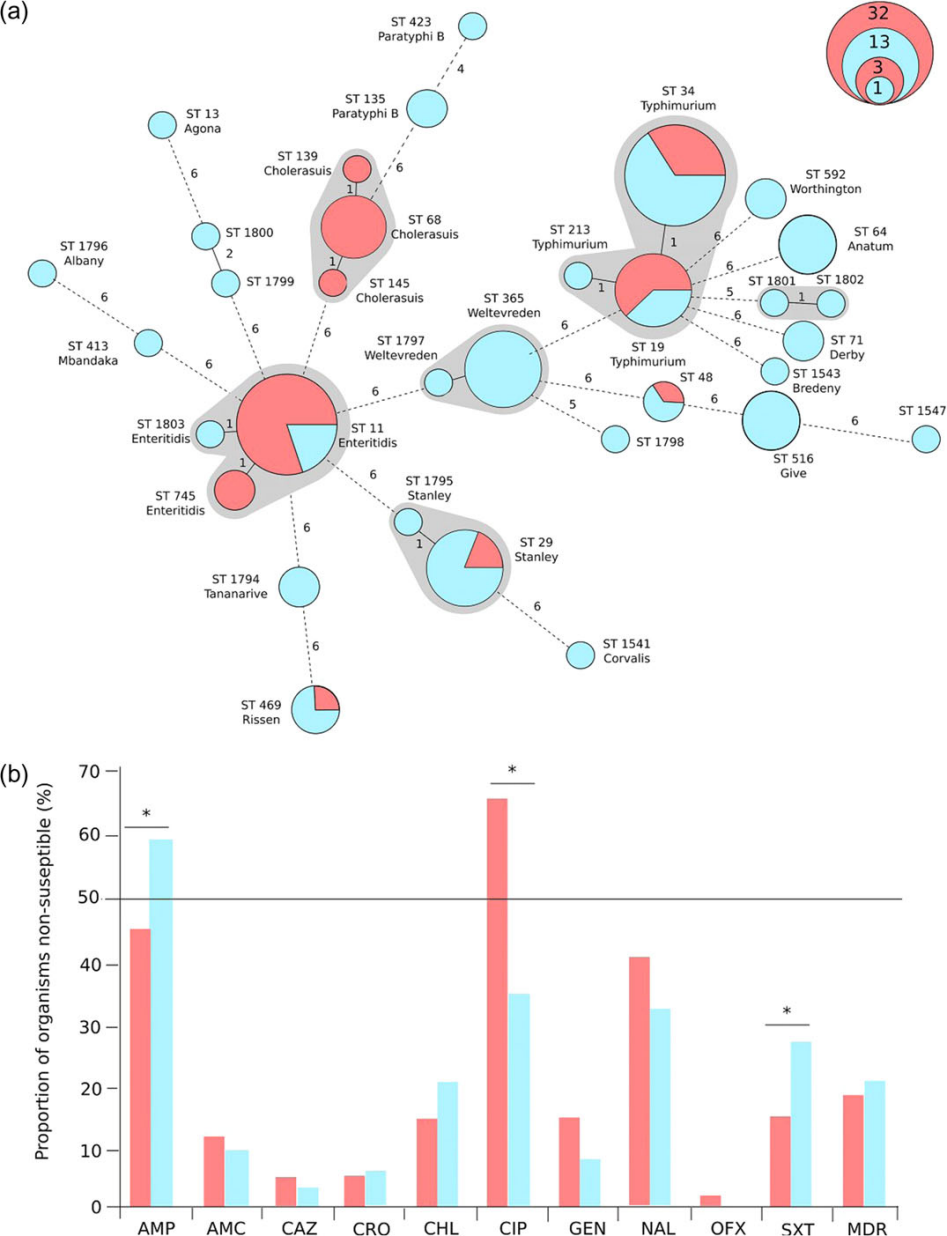
Identified *Salmonella* serovars causing invasive and non-invasive disease in Lao and their antimicrobial susceptibility profiles. (a) Minimum spanning tree of 168 Laotian NTS isolates created using seven-allele MLST profiling; the sources of the organisms are colour coded (red, blood; blue, faeces). The sequence type (ST) of each allele profile is shown along with the inferred serovar. Clonal complexes (*S*. *enterica* Typhimurium, *S. enterica* Enteritidis, *S*. *enterica* Choleraesuis, *S*. *enterica* Stanley and *S*. *enterica* Weltevreden) are highlighted. The size of each ST group corresponds with the number of isolates with the same ST profile (scale shown) and the branches are labelled by the number of variable alleles between STs. (b) Bar graph showing the proportion of organisms (red, blood; blue, faeces) exhibiting non-susceptibility (including intermediate resistance) against ampicillin (AMP), amoxicillin/clavulanate (AMC), ceftazidime (CAZ), ceftriaxone (CRO), chloramphenicol (CHL), ciprofloxacin (CIP), gentamicin (GEN), nalidixic acid (NAL), ofloxacin (OFX) and trimethoprim-sulfamethoxazole (SXT). Asterisks signify statistically significant differences in the proportion of organisms with non-susceptibility against the individual antimicrobial (p<0.05, Fisher’s exact test).

A total of 168 NTS isolates collected between 2006 and 2012 were available for characterization; 63 originated from blood and 105 from diarrhoeal faecal samples. iNTS disease-causing blood isolates were collected from patients admitted to Mahosot Hospital between 2000 and 2012 (n=53) and other hospitals in and around Vientiane (n=8). Two further isolates, collected in 2008 and 2010, originated from patients attending Luang Namtha Provincial Hospital in northwestern Lao and Saravan Provincial Hospital in southern Lao, respectively.^19^ DNA was extracted from all 168 blood and faecal isolates and subjected to PCR amplification for standard seven-allele MLST. The resulting minimum spanning tree of the MLST data, with organisms identified by sequence type (ST) and inferred serovar and categorized by sampling site, is shown in Figure 1a.

Among the six different serovars isolates from the blood, *S. enterica* Enteritidis (Group D; n=28), *S. enterica* Typhimurium (Group B; n=19) and *S. enterica* Choleraesuis (Group C1; n=11) were the most common, accounting for >90% (58/63) of the iNTS disease cases. In contrast, isolates from faecal samples collected from diarrhoeal patients were characterized by a broader range of NTS serogroups (n=6) and serovars (n=18; Figure 1a and Table 1). The mostly commonly identified serovars from non-invasive infections were *S. enterica* Typhimurium (n=28), *S. enterica* Weltevreden (n=14) and *S. enterica* Stanley (n=15).

**Table 1.** The distribution of NTS serovars isolated from the blood and faecal specimens of patients in Lao PDR

Serogroup^a^	*Salmonella* serovar^b^	Bloodstream isolates, n (%) (N=63)	Faecal isolates, n (%) (N=105)	p-Value^c^
B	Typhimurium	19 (30.2)	28 (26.7)	0.625
	Stanley	3 (4.8)	15 (14.3)	0.071
	Paratyphi B	0	5 (4.8)	0.158
	Derby	0	2 (1.9)	0.528
	Bredeney	0	1 (1.0)	1.000
	Agona	0	1 (1.0)	1.000
D	Enteritidis	28 (44.4)	7 (6.7)	**<0.001**
	Panama/Miami/Koessen	1 (1.6)	2 (1.9)	1.000
C1	Choleraesuis	11 (17.5)	0 (0)	**<0.001**
	Rissen	1 (1.6)	3 (2.9)	1.000
	Mbandaka	0	1 (1.0)	1.000
C2	Albany	0	1 (1.0)	1.000
	Corvallis	0	1 (1.0)	1.000
	Tananarive	0	3 (2.9)	1.000
E	Weltevreden	0	14 (13.3)	**0.001**
	Anatum	0	5 (7.5)	0.158
	Give	0	6 (5.7)	0.085
G	Worthington	0	2 (1.9)	0.528
C/E/G	Unknown	0	8 (7.6)	0.026

^a^Serogroup determined by classical O serotyping.

^b^Serovar inferred by MLST profile.

^c^p-Values determined by Fisher’s exact test on the proportions of the specific *Salmonella* serovar isolated from blood and faecal specimens. Significant p-values in bold.

To identify specific serovars significantly associated with invasive infections we compared the proportions of the various serovars between the syndromes (Table 1). Notably, the proportion of *S. enterica* Typhimurium isolated from the blood or faeces was not significantly different (p=0.27; Fisher’s exact test). In contrast, *S. enterica* Enteritidis and *S. enterica* Choleraesuis were significantly more associated with invasive disease than diarrhoeal infection in this patient group (p<0.001 for both comparisons). Conversely, *S. enterica* Weltevreden was significantly more associated with diarrhoea than systemic infection (p=0.001).

We measured the susceptibility of the 168 NTS isolates to 10 different antimicrobials by disc diffusion and categorized the data according to whether isolated from blood or faeces (Figure 1b). We found no significant difference in the proportion of susceptible or non-susceptible isolates cultured from each sample type for the majority (7/10) of antimicrobials tested. However, organisms isolated from faecal samples were significantly less likely to be susceptible to ampicillin and trimethoprim-sulfamethoxazole compared with blood isolates (p<0.05 for both comparisons). We additionally determined that 65% (41/63) of the bloodstream isolates were non-susceptible to ciprofloxacin, which was a significantly higher proportion than in faecal isolates (p<0.001). Notably, the majority of these *Salmonella* isolates were categorized as having intermediate resistance to ciprofloxacin, and only one isolate, a *S. enterica* Choleraesuis from a blood culture, tested fully resistant. This isolate was also resistant to the majority of other antimicrobials tested.

There are limited data regarding the presentation of patients with iNTS disease in Asia; clinical data for 62 iNTS disease patients are summarized in Table 2. Most patients were female (35/62 [56%]) with a median age of 32 y (interquartile range [IQR] 17–44), while the median duration of illness before hospital admission was 6 d (IQR 2–13). The most commonly reported symptoms were fever (>38.0°C [59/62 {95%}]), headache (40/62 [65%]) and diarrhoea (36/62 [58%]). Given the known association between iNTS disease and HIV infection, it was unexpected that only 6 of the 50 (12%) patients tested were HIV seropositive. Unsurprisingly in this endemic setting, 3/62 (5%) patients had pulmonary tuberculosis as an additional comorbidity.

**Table 2.** The clinical features of patients in Lao PDR with iNTS disease

Characteristic	Value
Sex (male)	27/62 (43.5)
Age (y), median (IQR)	32 (17–44)
Days ill before hospital, median (IQR)	6 (2–13)
Fever	59/62 (95.2)
Rigors	26/61 (42.6)
Headache	40/62 (64.5)
Arthralgia	24/60 (40.0)
Back pain	14/59 (23.7)
Myalgia	27/54 (50.0)
Jaundice	9/54 (16.7)
Nausea	19/61 (31.1)
Vomiting	22/62 (35.5)
Dysuria	4/60 (6.7)
Diarrhoea	36/62 (58.1)
Constipation	4/53 (7.5)
*Mycobacterium tuberculosis* infection	3/44 (6.8)
HIV seropositive	6/50 (12.0)
Antimicrobial taken in the last 7 d	15/41 (36.6)

All values are n/N (%) unless otherwise noted.

## Discussion

Invasive NTS is now well recognized as a major cause of community-acquired bloodstream infections in parts of sub-Saharan Africa,^7^ particularly in children and those infected with HIV.^20^ Outside of this setting, we and others have more recently described the additional burden of iNTS disease in Vietnam and Southeast Asia,^10,21^ which has previously remained unrecognized.^22^ The data presented here confirm that iNTS disease is also endemic in Lao and is most commonly caused by *S*. *enterica* Cholerasuis, *S. enterica* Typhimurium and *S*. *enterica* Enteritidis. Our data were collected from patients presenting to a hospital and routine laboratory testing, therefore, while we are unable to comment on the incidence of iNTS disease in Lao, we postulate that iNTS disease is less common in this setting than in parts of sub-Saharan Africa.

We performed MLST and molecular serotyping on 63 *Salmonella* isolated from blood and 105 *Salmonella* isolated from faecal samples. We identified a wide range of *Salmonella* in the faecal specimens, with the main serovars being *S. enterica* Typhimurium, *S*. *enterica* Weltevreden and *S. enterica* Stanley. Given the epidemiology and zoonotic transmission potential of NTS serovars, we suggest that this variation in the causative agents of *Salmonella* gastroenteritis is largely associated with circulation of these organisms in the food chain. *S. enterica* Derby, *S*. *enterica* Anatum, *S. enterica* Stanley and *S*. *enterica* Weltewreden have been previously isolated from slaughterhouse pigs in Vientiane, suggesting widespread circulation of these organisms in Lao.^14^*S. enterica* Weltevreden in particular is a common zoonotic infection in Southeast Asia and has been widely reported across the region.^11,13^ This organism is among the most common causes of *Salmonella*-associated diarrhoea in neighbouring Thailand and is thought to be associated with aquaculture.^23^*S. enterica* Stanley is also known to cause gastroenteritis in Southeast Asia, and was found to be associated with *Salmonella* gastroenteritis (24/26 cases) in Mahosot Hospital in Vientiane in the late 1960s.^24^ Our data confirm that *S. enterica* Stanley is still endemic and associated with diarrhoea at this location.

In contrast to the broad distribution of *Salmonella* serovars found in diarrhoeal faecal samples, the range of *Salmonella* isolated from blood was more limited, with only six different serovars (nine STs) identified. Internationally, *Salmonella* serovars Typhimurium, Enteritidis and Dublin are most commonly associated with iNTS disease. A particular well-reported issue in sub-Saharan Africa has been iNTS disease caused by *S. enterica* Typhimurium ST313, which is frequently multidrug resistant (MDR) and not commonly associated with diarrhoeal symptoms and is therefore more similar to typhoidal serovars in presentation.^5,25^ In our study, the most common *Salmonella* serovars isolated from blood were Enteritidis, Typhimurium and Cholerasuis. This distribution is almost identical to that which we recently described in Vietnam.^10^ Notably, we did not identify *S. enterica* Typhimurium ST313 but did isolate *S. enterica* Typhimurium ST19 and ST34. These STs have been described previously with invasive disease in Southeast Asia,^10^ and both were isolated from faecal specimens. In contrast, *S. enterica* Choleraesuis was exclusively associated with invasive disease; this serovar is known to circulate in pigs and has been associated with iNTS disease in other countries in Asia.^23,26^

The emergence of iNTS disease-causing organisms in sub-Saharan Africa with resistance to various antimicrobials, including chloramphenicol, ampicillin and co-trimoxazole, has been well described.^3,27^ Of concern are additional reports of resistance against third-generation cephalosporins.^6,28^ Here we found that resistance to ampicillin was common in both invasive and non-invasive organisms (>40%) and non-susceptibility against ciprofloxacin was more apparent in blood isolates than faecal isolates. This non-susceptibility against ciprofloxacin indicates the circulation of these organisms within the human population and the empirical use of fluoroquinolones for febrile disease at this location.^17^ The majority of the organisms were susceptible to third-generation cephalosporins, suggesting ceftriaxone to be an appropriate selection for therapy.

There are several limitations to our study. First, the organisms described were collected during routine care in specific health care facilities and are therefore unlikely to be representative of the entire distribution of *Salmonella* circulating in this Southeast Asia country. Second, faecal culture is not routinely performed in this setting and therefore sampled patients may not be representative of the full clinical or microbiological spectrum of infection. Lastly, as this study focussed on human infection, we were unable to assess the role of potential zoonotic transmission, which is likely to be significant in this setting.^15^ Despite these limitations, we describe the first data from Lao defining the *Salmonella* serovars associated with iNTS disease and their antimicrobial susceptibility profiles.

## Conclusions

We investigated the causative serovars of non-invasive and iNTS disease and their associated antimicrobial susceptibility profiles in a major tertiary hospital in Vientiane, Lao. We find a differing distribution of *Salmonella* STs and serovars associated with invasive and non-invasive disease but a comparable array of iNTS disease-causing serovars between Lao and neighbouring Vietnam. There is a small but not-insignificant burden of iNTS disease in Lao; further clinical and epidemiological investigations are required to assess mortality and the role of co-morbidities such as HIV.

## References

[1] Majowicz SE, Musto J, Scallan E, et al The global burden of nontyphoidal *Salmonella* gastroenteritis. Clin Infect Dis. 2010;50(6):882–9. 20158401 10.1086/650733

[2] Gordon MA *Salmonella* infections in immunocompromised adults. J Infect. 2008;56(6):413–22. 18474400 10.1016/j.jinf.2008.03.012

[3] Kingsley RA, Msefula CL, Thomson NR, et al Epidemic multiple drug resistant *Salmonella* Typhimurium causing invasive disease in sub-Saharan Africa have a distinct genotype. Genome Res. 2009;19(12):2279–87. 19901036 10.1101/gr.091017.109 PMC2792184

[4] Nga TVT, Parry CM, Le T, et al The decline of typhoid and the rise of non-typhoid salmonellae and fungal infections in a changing HIV landscape: bloodstream infection trends over 15 years in southern Vietnam. Trans R Soc Trop Med Hyg. 2012;106(1):26–34. 22137537 10.1016/j.trstmh.2011.10.004

[5] Feasey NA, Dougan G, Kingsley RA, et al Invasive non-typhoidal salmonella disease: an emerging and neglected tropical disease in Africa. Lancet. 2012;379(9835):2489–99. 22587967 10.1016/S0140-6736(11)61752-2 PMC3402672

[6] Lunguya O, Lejon V, Phoba M-F, et al Antimicrobial resistance in invasive non-typhoid *Salmonella* from the Democratic Republic of the Congo: emergence of decreased fluoroquinolone susceptibility and extended-spectrum beta lactamases. PLoS Negl Trop Dis. 2013;7(3):e2103. 23516651 10.1371/journal.pntd.0002103 PMC3597487

[7] Kariuki S, Revathi G, Kariuki N, et al Invasive multidrug-resistant non-typhoidal Salmonella infections in Africa: zoonotic or anthroponotic transmission? J Med Microbiol. 2006;55:585–91. 16585646 10.1099/jmm.0.46375-0

[8] Langridge GC, Fookes M, Connor TR, et al Patterns of genome evolution that have accompanied host adaptation in *Salmonella*. Proc Natl Acad Sci USA. 2015;112(3):863–8. 25535353 10.1073/pnas.1416707112 PMC4311825

[9] Sinwat N, Angkittitrakul S, Coulson KF, et al High prevalence and molecular characteristics of multidrug-resistant *Salmonella* in pigs, pork and humans in Thailand and Laos provinces. J Med Microbiol. 2016;65(10):1182–93. 27542886 10.1099/jmm.0.000339

[10] Phu Huong Lan N, Le Thi Phuong T, Nguyen Huu H, et al Invasive non-typhoidal *Salmonella* infections in Asia: clinical observations, disease outcome and dominant serovars from an infectious disease hospital in Vietnam. PLoS Negl Trop Dis. 2016;10(8):e0004857. 27513951 10.1371/journal.pntd.0004857 PMC4981332

[11] Noor Uddin GM, Larsen MH, Barco L, et al Clonal occurrence of *Salmonella* Weltevreden in cultured shrimp in the Mekong Delta, Vietnam. PLoS One. 2015;10(7):e0134252. 26222547 10.1371/journal.pone.0134252 PMC4519254

[12] Ponce E, Khan AA, Cheng CM, et al Prevalence and characterization of *Salmonella enterica* serovar Weltevreden from imported seafood. Food Microbiol. 2008;25(1):29–35. 17993374 10.1016/j.fm.2007.09.001

[13] Makendi C, Page AJ, Wren BW, et al A phylogenetic and phenotypic analysis of *Salmonella enterica* serovar Weltevreden, an emerging agent of diarrheal disease in tropical regions. PLoS Negl Trop Dis. 2016;10(2):e0004446. 26867150 10.1371/journal.pntd.0004446 PMC4750946

[14] Boonmar S, Markvichitr K, Chaunchom S, et al *Salmonella* prevalence in slaughtered buffaloes and pigs and antimicrobial susceptibility of isolates in Vientiane, Lao People’s Democratic Republic. J Vet Med Sci. 2008;70(12):1345–8. 19122403 10.1292/jvms.70.1345

[15] Boonmar S, Morita Y, Pulsrikarn C, et al *Salmonella* prevalence in meat at retail markets in Pakse, Champasak Province, Laos, and antimicrobial susceptibility of isolates. J Glob Antimicrob Resist. 2013;1(3):157–61. 27873626 10.1016/j.jgar.2013.05.001

[16] Phetsouvanh R, Phongmany S, Soukaloun D, et al Causes of community-acquired bacteremia and patterns of antimicrobial resistance in Vientiane, Laos. Am J Trop Med Hyg. 2006;75(5):978–85. 17124000 PMC2213713

[17] Clinical and Laboratory Standards Institute Performance standards for antimicrobial susceptibility testing: twenty-fourth informational supplement. Document no. M100-S24. Wayne, PA: Clinical and Laboratory Standards Institute, 2014.

[18] Achtman M, Wain J, Weill FX, et al Multilocus sequence typing as a replacement for serotyping in *Salmonella enterica*. PLoS Pathog. 2012;8(6):e1002776. 22737074 10.1371/journal.ppat.1002776 PMC3380943

[19] Mayxay M, Castonguay-Vanier J, Chansamouth V, et al Causes of non-malarial fever in Laos: a prospective study. Lancet Glob Health. 2013;1(1):e46–54. 24748368 10.1016/S2214-109X(13)70008-1 PMC3986032

[20] Gilchrist JJ, MacLennan CA, Hill AVS Genetic susceptibility to invasive *Salmonella* disease. Nat Rev Immunol. 2015;15(7):452–63. 26109132 10.1038/nri3858

[21] Southeast Asia Infectious Disease Clinical Research Network Causes and outcomes of sepsis in southeast Asia: a multinational multicentre cross-sectional study. Lancet Glob Health. 2017;5(2):e157–67. 28104185 10.1016/S2214-109X(17)30007-4 PMC5332551

[22] Ao TT, Feasey NA, Gordon MA, et al Global burden of invasive nontyphoidal *Salmonella* disease, 2010. Emerg Infect Dis. 2015;21(6):941–9. 10.3201/eid2106.140999 PMC4451910 25860298

[23] Sirichote P, Bangtrakulnonth A, Tianmanee K, et al Serotypes and antimicrobial resistance of *Salmonella enterica* ssp in central Thailand, 2001–2006. Southeast Asian J Trop Med Public Health. 2010;41(6):1405–15. 21329317

[24] Dujeu G [Salmonella stanley in Vientiane]. Med Trop (Mars). 1970;30(2):167–70. 5426016

[25] Okoro CK, Barquist L, Connor TR, et al Signatures of adaptation in human invasive *Salmonella* Typhimurium ST313 populations from sub-Saharan Africa. PLoS Negl Trop Dis. 2015;9(3):e0003611. 25803844 10.1371/journal.pntd.0003611 PMC4372345

[26] Chiu C, Su L, Chu C *Salmonella enterica* serotype Choleraesuis: epidemiology, pathogenesis, clinical disease, and treatment. Clin Microbiol Rev. 2004;17(2):311–22. 15084503 10.1128/CMR.17.2.311-322.2004 PMC387403

[27] Gordon MA, Graham SM, Walsh AL, et al Epidemics of invasive *Salmonella enterica* serovar Enteritidis and *S. enterica* serovar Typhimurium infection associated with multidrug resistance among adults and children in Malawi. Clin Infect Dis. 2008;46(7):963–9. 18444810 10.1086/529146

[28] Mahon BE, Fields PI Invasive Infections with Nontyphoidal Salmonella in Sub-Saharan Africa Microbiol Spectr. 2016 Jun;4(3). 10.1128/microbiolspec.EI10-0015-2016 27337467

